# Low spinophilin expression enhances aggressive biological behavior of breast cancer

**DOI:** 10.18632/oncotarget.3586

**Published:** 2015-03-14

**Authors:** Daniela Schwarzenbacher, Verena Stiegelbauer, Alexander Deutsch, Anna Lena Ress, Ariane Aigelsreiter, Silvia Schauer, Karin Wagner, Tanja Langsenlehner, Margit Resel, Armin Gerger, Hui Ling, Cristina Ivan, George Adrian Calin, Gerald Hoefler, Beate Rinner, Martin Pichler

**Affiliations:** ^1^ Division of Oncology, Department of Internal Medicine, Medical University of Graz, Austria; ^2^ Division of Hematology, Department of Internal Medicine, Medical University of Graz, Austria; ^3^ Institute of Pathology, Medical University of Graz, Austria; ^4^ Center for Medical Research, Medical University of Graz, Austria; ^5^ Department of Therapeutic Radiology and Oncology, Medical University of Graz, Austria; ^6^ Department of Experimental Therapeutics, The University of Texas MD Anderson Cancer Center, TX, USA; ^7^ Center for RNA Interference and Non-Coding RNAs, The University of Texas MD Anderson Cancer Center, TX, USA

**Keywords:** breast cancer, tumor suppressor, prognosis, cellular growth, invasion

## Abstract

Spinophilin, a putative tumor suppressor gene, has been shown to be involved in the pathogenesis of certain types of cancer, but its role has never been systematically explored in breast cancer. In this study, we determined for the first time the expression pattern of spinophilin in human breast cancer molecular subtypes (n = 489) and correlated it with survival (n = 921). We stably reduced spinophilin expression in breast cancer cells and measured effects on cellular growth, apoptosis, anchorage-independent growth, migration, invasion and self-renewal capacity *in vitro* and metastases formation *in vivo*. Microarray profiling was used to determine the most abundantly expressed genes in spinophilin-silenced breast cancer cells. Spinophilin expression was significantly lower in basal-like breast cancer (*p*<0.001) and an independent poor prognostic factor in breast cancer patients (hazard ratio = 1.93, 95% confidence interval: 1.24-3.03; *p* = 0.004) A reduction of spinophilin levels increased cellular growth in breast cancer cells (*p*<0.05), without influencing activation of apoptosis. Anchorage-independent growth, migration and self-renewal capacity *in vitro* and metastatic potential *in vivo* were also significantly increased in spinophilin-silenced cells (*p*<0.05). Finally, we identified several differentially expressed genes in spinophilin-silenced cells. According to our data, low levels of spinophilin are associated with aggressive behavior of breast cancer.

## INTRODUCTION

Breast cancer (BC) is the most frequently diagnosed cancer and the leading cause of cancer related death among women [1]. According to the American Cancer Society it is estimated that currently around 234,580 people are diagnosed with BC and 40,030 are dying of the disease in the United States [2]. Despite novel treatment modalities combining anti-cancer drugs, surgery and radiotherapy, metastatic BC remains an incurable disease [3]. BC is highly heterogeneous and can be sub-classified into different molecular subtypes including luminal A, luminal B, basal-like and HER2-enriched BC [4, 5]. These molecular subtypes can be considered as different diseases in terms of biological behavior, prognosis and treatment schedule. Especially the basal-like subtype has been proven very aggressive, without any currently approved specific targeted therapeutic agent [6]. Therefore, identification of underlying molecular cancer driving factors is of utmost importance to discover potentially novel therapeutic avenues for BC patients.

Spinophilin (also known as Neurabin 2 or PPP1R9B) is a multifunctional scaffold protein and a regulatory subunit of phosphatase 1a (PP1a) whose gene is located at the chromosomal region 17q21.33 [7]. Spinophilin knockout-mice displayed early appearance of tumors, a reduced lifespan as well as increased cellular proliferation in mammary ducts [8]. In several other types of cancer including hepatocellular carcinoma, lung, head and neck and colorectal cancer, a reduced or loss of spinophilin expression and its association with poor prognostic factors have been described [9-12]. The previously shown influence on proliferative capacity in some cancers indicates to a potential tumor suppressive role of this protein [7].

In human BC, the role of spinophilin has never been systematically explored yet. Therefore, we analyzed the spinophilin expression data of 921 BC patients available from The Cancer Genome Atlas (TCGA) dataset and correlated spinophilin expression with different molecular subtypes and survival. Subsequently, based on the association of low spinophilin levels with basal-like BC and poor prognosis, we silenced spinophilin expression in human breast cancer cell lines and examined the effects of reduced spinophilin expression on different parameters of biological properties in BC.

## RESULTS

**Figure 1:**
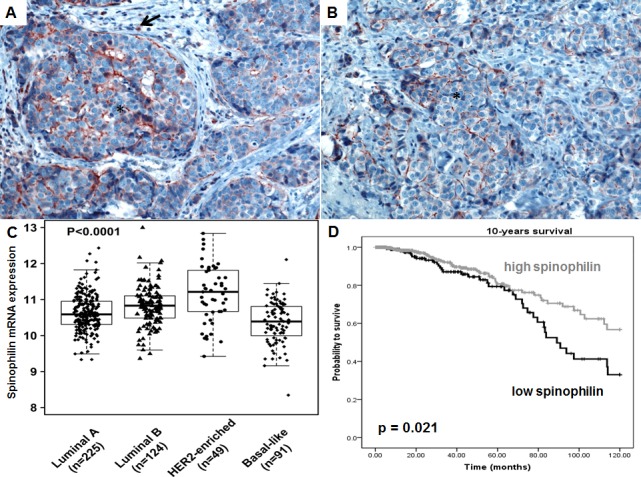
Spinophilin expression in breast cancer tissue and different molecular subtypes (A-B) A strong, membranous staining pattern could be observed in breast cancer cells (*) in tissue slides of breast cancer patients. Surrounding inflammatory cells are also positively stained (arrow). (C) Analysis of 489 breast cancer patients of the TCGA data set indicates that basal-like breast cancer subtype exhibit the lowest spinophilin expression. (D) In 921 breast cancer patients, a low spinophilin level is significantly associated with poor survival.

**Figure 2:**
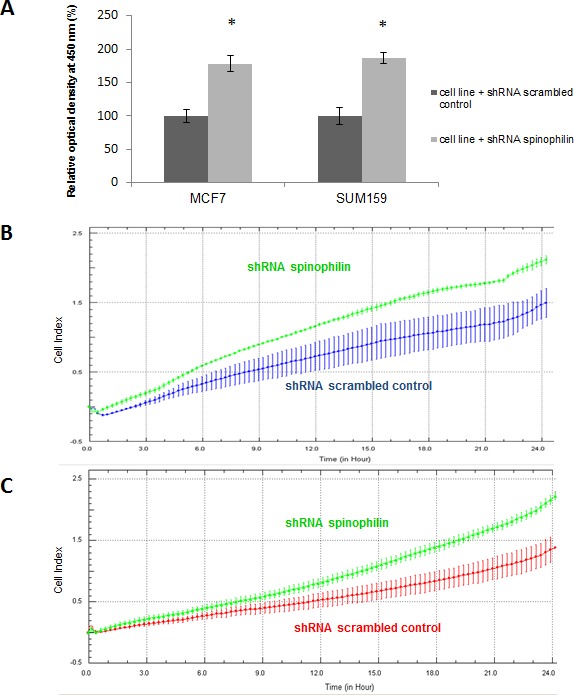
Silencing of spinophilin increases cellular growth rates, migration and invasion (A) A significant increase (*p*<0.05) of cell growth has been observed in both cell lines with reduced spinophilin levels. Data are generated by three independent biological replicates with six technical replicates using the WST-1 assay. (B) The silencing of spinophilin in SUM159 cells promotes cell migration in the xCELLigence system compared to control cells as indicated by the increase of the cell index on the Y-axis. (C) The silencing of spinophilin in SUM159 cells also leads to an increased invasion in the xCELLigence system compared to control cells as indicated by the increase of the cell index. Plotted curves represent the averages from two independent wells/measurements and cells were monitored for 24 hours.

At first we examined the localization and tissue distribution of spinophilin expression by immunohistochemistry in human BC samples. A strong, membranous staining pattern with a varying frequency of positivity could be observed in BC cells in the context of the cancer tissue. In the surrounding stroma tissue, inflammatory cells and endothelial cells were also positively stained for spinophilin (Figure 1A, B). After confirming the expression in BC cells, we explored differences of spinophilin mRNA expression levels between different BC molecular subtypes (i.e. luminal A, luminal B, Her2-enriched and basal-like) on a large scale RNA seq data set of 489 BC patients (TCGA data set). Basal-like BC tissue showed the lowest spinophilin mRNA expression levels when compared to other BC subtypes (*p*<0.001, Figure 1C). Furthermore, in 921 patients with available survival data, low spinophilin expression level was associated with poor survival (*p* = 0.021, Figure 1D). Multivariate Cox analysis including age, tumor stage, estrogen receptor status, Her2/neu receptor status and spinophilin expression confirmed low levels of spinophilin as an independent prognostic factor in BC patients (hazard ratio: 1.93, 95% confidence interval 1.24-3.03; *p* = 0.004). In addition to spinophilin levels, age, tumor stage and negative hormone receptor status were independent prognostic factors (*p* <0.001 for all parameters). To further characterize the biological role of spinophilin expression in BC cells, we used a shRNA lentiviral vector system to transduce and silence spinophilin. We selected SUM159 cells as a basal-like cell line model and MCF-7 cells as a luminal A cellular model [13]. SUM159 cells are p53 gene mutated, whereas MCF-7 cells are p53 wild-type cells [14]. Analogous to the TGCA patient data, SUM159 cells have naturally occurring lower spinophilin levels than MCF-7 cells in qRT-PCR and Western Blot analysis (Supplementary Figure 1A and 1B). Using shRNA, a silencing effect was confirmed by reduced spinophilin protein levels in Western Blot analyses for both cell lines (Supplementary Figure 2). Subsequently, we explored the effects of reduced spinophilin expression on cellular growth rates of these cell lines. A significantly increased cellular growth could be detected in spinophilin silenced MCF-7 cells (78% increase ±12%, *p*<0.05) and in SUM159 cells (86% increase ±8%, *p*<0.05, Figure 2A) compared to the control cells. To substantiate these findings with a second independent method we monitored cellular growth rates by the xCELLigence system. This real-time growth assay continuously detects the well impedance as a measure of cell density. As shown in Supplementary Figure 3, cellular growth, as indicated by the cell index on the Y-axis, also increases in cells with reduced spinophilin expression compared to control cells.

Next, to exclude that differences in apoptotic activity in the spinophilin-silenced cells influence the growth assays, we measured the gene expression of the pro-apoptotic BAX (BCL2-associated X protein) and the anti-apoptotic Bcl-2 (B-cell CLL/lymphoma 2) gene, but detected no significant differences (Supplementary Figure 4). To confirm this observation with a second method, we used Western blot to detect PARP (Poly ADP ribose polymerase) cleavage as a marker for increased apoptosis. In Western blot analysis, we could only detect the full length (119 kDa) PARP form, whereas cleaved PARP (85 kDa) as a marker for increased apoptosis was not detected (Supplementary Figure 2). Independent of apoptosis analyses, for phosphorylated retinoblastoma protein we found no significant difference with regard to activation of this protein (data not shown).

**Figure 3:**
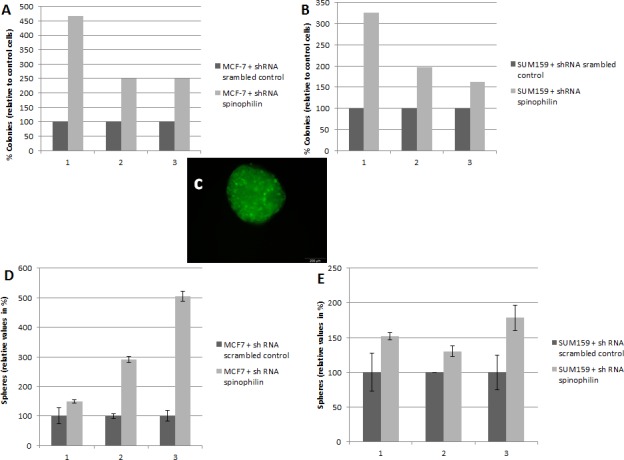
Silencing of spinophilin increases anchorage-independent growth and tumor sphere formation Graphs represent the results from three independent biological replicates of the breast cancer cell lines MCF-7 (A) and SUM159 (B) stably transfected with shRNA against spinophilin compared to control cells. In each biological replicate, a significantly increased number of colonies were observed in the spinophilin-silenced cells. (C) A representative example of a tumor sphere (mammosphere) under ultra-low attachment conditions. The transfected cells are labeled with green-fluorescent protein. Significant increase in the number of mammospheres after spinophilin-silencing in MCF-7 (D) and SUM159 (E) cells.

Silencing of spinophilin in SUM159 cells also promotes cell migration (Figure 2B) and invasion (Figure 2C) compared to control cells. We also measured the epithelial-mesenchymal transition-related markers e-cadherin and vimentin, but could not detect any significant differences in mRNA levels (data not shown). We further investigated a possible role of spinophilin expression on soft agar and mammosphere assay. The mammosphere assay is widely used for the quantification of stem cell self-renewal capacity, whereas the soft agar assay is commonly used to assess anchorage- independent growth. In the soft agar assay, a significantly higher (*p*<0.05) number of colonies in spinophilin-silenced cells compared to control cells for both cell lines and all three independent biological replicates were observed (Figure 3A, B).

**Figure 4:**
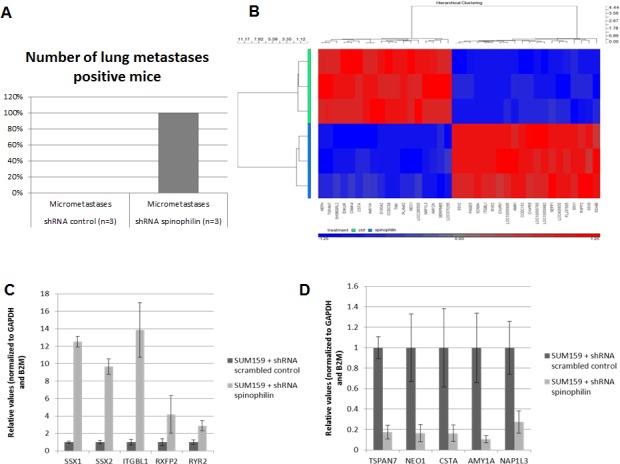
*In vivo* metastases formation and gene expression profile in spinophilin-silenced basal-like breast cancer cells (A) Shows the number of mice positively explored for lung micrometastases in the SUM159 xenografts (B): HeatMap of the top differentially expressed genes of SUM159 with silenced spinophilin compared to control cells. Genes clustered using hierarchical clustering (Pearson's dissimilarity, Ward’ method) on expression values present in picture. Genes shifted to mean of zero and scaled to standard deviation of one. Five up- (C) and five down-regulated genes (D) were selected and validated by qRT-PCR.

Consequently, we investigated the effect of spinophilin-silencing on the self-renewal capacity by using mammosphere assays (Figure 3C). Again for both cell lines, the number of mammospheres were significantly higher (*p*<0.05) in spinophilin-silenced cells (Figure 3D, E). To confirm these aggressive biological *in vitro* features *in vivo*, we evaluated the metastatic potential in mice that were subcutaneously injected with spinophilin-silenced SUM159 cells. After 16 days of observation, 100% of mice with spinophilin-silenced cells showed micrometastases in the lungs, whereas none of the control mice showed signs of lung metastases (Figure 4A, *p* < 0.05).

After identifying that low spinophilin expression is associated with aggressive biological behavior in BC cells, we further tried to figure out which genes are most differentially up- or down-regulated in spinophilin-silenced BC cells. Therefore, we performed microarray gene expression analysis in three independent biological replicates comparing SUM159 spinophilin-silenced and control cells. Most important changes of gene expression are shown in the Heat map in Figure 4A and a list of the 30 top up- and down-regulated genes as well as a pathway analysis is included in Supplementary Table 2 and 3. Consequently, the five most up- and down-regulated protein-coding genes were further validated using quantitative RT-PCR. A 100% concordance between microarray results and the confirmatory RT-PCR was found. Under the differentially expressed genes we identified several genes previously related to cancer including the up-regulated *SSX1*, *SSX2*, *RXFP2*, *RYR2* and the down-regulated *CSTA*, *TSPAN7*, *NEO1*, *S100A*, *SERPINB5* and *SEPP1* (Figure 4B, C). Using the expression data of the 921 BC patients of the TCGA dataset, we confirmed for some of these differentially expressed genes including *ITGBL1*, *TSPAN7* and *SERPINB5*, a significant association with spinophilin expression by using non-parametric tests (*p* < 0.05).

## DISCUSSION

Spinophilin is a protein phosphatase 1 binding protein that has been initially described in dendritic spines of the nervous system [7]. Previous studies reported that spinophilin is important for cell-cell adhesion and functions as a link between the actin cytoskeleton and the plasma membrane [15-17]. Basically discovered to be essential for several processes in the nervous system, spinophilin has later been associated with certain types of cancer. Vivo and colleagues were the first who reported a connection between the human tumor suppressor protein ARF and spinophilin [18]. More recently published studies confirmed a role for spinophilin in human cancer. For instance, spinophilin expression had an inhibitory effect on anchorage-independent growth of glioblastoma cells [18, 19] as well as an effect on self-renewal and differentiation in brain tumor stem cells [20]. Molino-Pinelo and colleagues revealed that spinophilin expression correlates with higher grade of malignancy in lung cancers [9]. In hepatocellular carcinoma reduced levels of spinophilin have been associated with high proliferation and poor prognosis [10]. Another recent study suggests that down-regulation of spinophilin in colorectal cancer correlates with a more aggressive histologic phenotype, faster relapse and poorer survival in advanced stages of colorectal carcinoma [12]. Ress et al. confirmed the role of spinophilin in colorectal cancer and showed that reduced spinophilin levels led to increased cellular growth rates, anchorage-independent growth [21]. Interestingly, abnormalities in the growth of mammalian ducts have been observed in spinophilin knock-out mice [7].

In the present study, which is the first one investigating the role of spinophilin in BC, we observed a membranous localization of spinophilin in BC cells. This finding is in concordance with its role as a scaffold protein linking the cell membrane with the cytoskeleton [15]. Furthermore, in a large external cohort of BC samples, we found that lower spinophilin expression is significantly associated with basal-like BC and poor prognosis. Basal-like BC is the most aggressive subtype showing high rates of proliferation. Up to now cytotoxic chemotherapeutic agents are the only approved treatment option [6], which makes the discovery of new cancer-driving factors of paramount interest. By generating stable spinophilin-silenced cell lines, we observed higher cellular growth rates in basal-like and luminal A BC cells, both with different p53 mutational status. These findings suggest that spinophilin increases cellular growth rates in BC cells regardless of the underlying molecular subtype or p53 mutations. These results are also in line with previous studies in different types of cancer including colorectal and hepatocellular carcinoma. These studies also clearly demonstrated that a loss of spinophilin leads to a proliferation promoting effect, regardless to the p53 mutation status [10, 12]. In addition to proliferation, we also found that reduced levels of spinophilin triggersmigration and anchorage-independent cell growth. However, cell migration and invasion assays can also be influenced by varying cell numbers due to proliferation differences. Mammosphere assays, which are frequently used to confirm the self-renewal capacity of putative BC stem cells [22, 23], also demonstrated an increase in sphere formation capacity of spinophilin-silenced cells. The use of xenografts confirms the increased metastatic potential in spinophilin-silenced cells *in vivo*. Besides the role as a scaffold protein, spinophilin has been described as a multifunctional protein interacting with several other proteins. These promiscuous molecular functions make the identification of the direct underlying mechanisms more difficult. In an attempt to identify at least some differentially expressed genes which might interact or act as down-stream affected genes, microarray analysis identified several interesting candidates. On the one hand the up-regulated genes include the gene family sister-of-Sex-lethal (*SSX*), which is considered as a multi-gene family consisting of 9 complete genes on chromosome Xp11. Among normal tissues its expression is restricted to testis, but it has been described in a variety of human tumors [24]. For instance, expression of *SSX2* has been associated with metastatic prostate cancer [25], advanced tumor stage and malignant tumors [26]. Interestingly, *SSX* gene expression has also been described previously in BC. Overexpression of *SSX2* in the typically low-invasive BC cell line MCF-7 induced cellular growth and promoted cell invasion [27]. RXFP2 (relaxin/insulin-like family peptide receptor 2) is one of the RXFP receptors for relaxin. As relaxin acts as a growth factor, this indicates a possible role of relaxin in cancer biology [28]. RXFP2 is also a receptor for INSL3 (insulin-like peptide 3) and has been implicated with tumor-promoting activity in thyroid cancer [29]. The receptor and its ligands have also been associated with cancer progression and invasiveness [30]. The ryanodine receptor (RyR, three isoforms) is a large, intracellular calcium channel and a recently published study demonstrated a mechanistic link between spinophilin and RyR2 activation in heart disease [31]. This calcium channel has also been connected to prostate [32] and breast cancer [33]. Abdul et al found a correlation between RyR expression and tumor grade in BC [34]. On the other hand, several down-regulated genes in the spinophilin-silenced cells could be identified. Cystatin A or stefin A (CSTA) is a cysteine protease inhibitor. A previous study showed a link between CSTA and metastasis in BC [35]. Later CSTA was also associated with the regulation of the progression of ductal carcinoma in situ to invasive BC. This study suggests a function of CSTA to normally suppress progression in BC [36]. The proteins of the tetraspanin superfamily are involved in cell motility, metastasis, cell proliferation and differentiation [37]. NEO1 (neogenin1) is a DCC-like (Deleted in Colorectal Cancer) netrin receptor and is significantly reduced in prostate tumors compared to normal prostate tissues [38]. S100A (S100 calcium binding protein A2) has been proposed to act as a tumor suppressor. S100A is down-regulated in numerous tumor types including BC. Knock-down of S100A2 in non-tumorigenic cells resulted in enhanced proliferation [39].

SERPINB5 (serpin peptidase inhibitor, clade B (ovalbumin), member 5) is implicated as a tumor suppressor which is absent in breast and prostate cancer [40] and has also a possible causal role in metastasis [41]. SEPP1 (selenoprotein P, plasma, 1) has been associated with breast cancer risk among women with higher Native American ancestry [42].

In conclusion, in this study we describe for the first time spinophilin expression in BC and link low expression levels to the aggressive basal-like BC. Apart the association, in a series of experiments we also found that BC cells with reduced spinophilin levels transform to a more aggressive biological phenotype. Future studies are warranted to further clarify the molecular mechanisms and biological role of spinophilin in BC patients. As breast cancer is a heterogeneous disease in terms of prognosis and treatment modalities, novel identified pathogenesis-driving factors might be helpful as novel prognostic markers or therapeutic targets.

## MATERIALS AND METHODS

### Immunohistochemistry

For measuring the localization and distribution of the spinophilin protein in BC tissue, we included eight formalin-fixed paraffin embedded BC cases from the Institute of Pathology, Medical University of Graz, Austria. The ethics committees of the Medical University of Graz approved this study (No. 24-248 ex 11/12). Immunohistochemical analysis for spinophilin expression was performed on whole tissue slides of BC tissue. In detail, the 3μm thick sections were deparaffinized in xylene and rehydrated with graded ethanol. For spinophilin detection, the sections were subjected to antigen retrieval in a pressure cooker (Dako, Pascal) in 0.01 M sodium-citrate buffer, pH 6.0, and subsequently incubated for 60 minutes with a rabbit antibody to human spinophilin (Millipore, Bedford, Massachusetts, USA; Anti-Spinophilin Antibody: AB5669) at a 1:50 dilution. The reaction was visualized using the UltraVision LP Large Volume Detection System HRP Polymer (Thermo Scientific, Rockford, IL) and all sections were counterstained with hematoxylin. For the negative control, the primary antibody was omitted. The localization and distribution of spinophilin-staining were evaluated by an experienced pathologist (A.A).

### Spinophilin mRNA expression in breast cancer subtypes and survival analysis

We downloaded and analyzed data (level 3 RNASeq v2) publicly available from the Cancer Genome Atlas Project (TCGA; http://tcga-data.nci.nih.gov/) for 921 BC patients. For 489 patients PAM50 molecular sub-classification results were acquired from the paper Comprehensive molecular portraits of human breast tumors [43]. Analyses were carried out in R statistical environment (version 3.0.1) (http:///www.r-project.org/). All tests were two-sided and considered statistical significant at the 0.05 level.

We checked for a relationship between spinophilin mRNA expression and overall survival as follows: Patients were grouped into percentiles according to spinophilin expression. The log-rank test was employed to determine the association between mRNA expression and overall survival. The Kaplan-Meier method was used to generate overall 10-years survival curves. The cut-off to optimally separate the patients into low/high spinophilin (log-rank test p-value minimum) was chosen (cut-off=0.26). Multivariate Cox proportional regression analyses were performed to determine the influence of patients' age (continuous variable), tumor stage (stage I, II, II and IV), hormone receptor (estrogen receptor positive versus negative) and Her2/neu receptor (Her2/neu positive versus negative/not available) status as well as spinophilin (dichotomized according to the above mentioned cut off) 10-years survival. Hazard ratios (HR's) estimated from Cox models were reported as relative risks with corresponding 95% confidence intervals (95%CI's). The samples were grouped into quartiles according to the score. The Shapiro-Wilk test was applied and verified that spinophilin expression does not follow a normal distribution in each PAM50 group. Accordingly, the nonparametric test Kruskal-Wallis test together with Nemenyi post-hoc test was applied to assess the relationship between spinophilin expression and PAM.50 subtype. A box-and-whisker plot (Box plot represents first (lower bound) and third (upper bound) quartiles, whiskers represent 1.5 times the interquartile range) was used to visualize the data (log2(x+1)).

### Breast cancer cell lines

The luminal A breast cancer cell line MCF-7 was purchased from American Type Culture Collection (ATCC) and the basal-like cell line SUM159 was obtained from Asterand (Detroid, MI). Identity of these cell lines was confirmed by STR analysis. MCF-7 cells were grown in MEM with Earle's salts containing 2 mmol/L L-glutamine (PAA, Pasching, Austria), 1% sodium pyruvate (PAA), 1% penicillin/streptomycin (PAA) and 10% FCS (PAA). SUM159 cells were maintained in Ham`s F12 containing 2 mmol/L L-glutamine (PAA), 2 mmol/L HEPES buffer (Gibco, Darmstadt, Germany), 5μg/ml insulin actrapid (Novo Nordisk, Vienna, Austria), 1μg/ml hydrocortisone (Sigma-Aldrich, Vienna, Austria) and 5% FCS (PAA). After obtaining a confluence of approximately 80%, total RNA was isolated following a standard Trizol protocol and RNA was stored at −80°C until further procedures.

### shRNA lentiviral particles transduction

SUM159 and MCF-7 cells were seeded in 6-well plates 24 hours prior to viral infection and incubated overnight in complete growth medium. On the day of transfection, the medium was replaced with complete growth medium containing 8 μg/ml polybrene (Santa Cruz Biotechnology, Santa Cruz, CA) and 10 μl of ViralPlus Transduction Enhancer (ABM, Richmond, BC, Canada). Cells were infected by adding 50 μl of shRNA spinophilin lentiviral particles (ABM, Sense strand: 5′-GGGAGGUGCGCAAGAUUAATT-3′, Antisense strand: 5′-UUAAUCUUGCGCACCUCCCGG-3′) or shRNA scrambled control lentiviral particles (ABM), respectively. Stably transfected SUM159 cells were selected with 0.5 μg/ml (MCF-7) or 1 μg/ml (SUM159) puromycin dihydrochloride (Gibco, Carlsbad, CA).

### Quantitative RT-PCR

For detection of mRNA expression levels after stable transfection experiments, 1 μg of total RNA was reverse transcribed by using QuantiTect Reverse Trascription Kit (Qiagen, Hilden, Germany) according to the manufacturer's protocol. Quantitative RT-PCR was carried out in technical duplicates of biological triplicates using commercially available primers specific for spinophilin (Hs_PPP1R9B_1_SG QuantiTect primer assay, Qiagen) and EMT-related genes (E-cadherin, Hs_CDH1_1_SG QuantiTect primer assay, Qiagen; Vimentin, Hs_VIM_1_SG QuantiTect primer assay, Qiagen). Primer sequences specific for Bcl-2, Bax, GAPDH, B2M, SSX1, SSX2, ITGBL1, RXFP2, RYR2, TSPAN7, NEO1, CSTA, AMY1A and NAP1L3 are listed in Supplementary Table 1. Quantitative RT-PCR was done on a LightCycler® 480 Real-Time PCR System (Roche Diagnostics, Mannheim, Germany) using the QuantiTect SYBR Green PCR Kit (Qiagen) according to the manufacturer's standard protocol. The arithmetic mean of the housekeeping genes GAPDH and B2M was used for normalization and relative gene expression levels were calculated using a standard 2^−ΔΔCT^ method [44]. Each experiment was performed in three independent biological replicates.

### Protein extraction and western blot analysis

Total proteins from stably transfected MCF-7 and SUM159 cells were extracted with radioimmunoprecipitation assay (RIPA) buffer (150 mM NaCl, 50 mM Tris-HCl, pH 7.5, 1% Triton, 0.1% SDS, 0.1% sodium deoxycholate and 1% Nonidet P40). 25 μg of total cellular proteins were resuspended in laemmli buffer (4% SDS, 20% glycerol, 10% 2-mercaptoethanol, 0.004% bromphenol blue and 0.125 M Tris HCl, pH approx. 6.8) and heated at 95 °C for 5 minutes. Proteins were separated by a 4–15% Mini-PROTEAN^®^ TGX^™^ Precast Gel (Biorad, Hercules, CA) and transferred onto a nitrocellulose membrane (Applichem, St. Louis, MO). The membrane was blocked for 1 hour with 3% non-fat dry milk in Tris buffered Saline/0.1% Tween-20. Immunoblotting was performed and antibodies specific for spinophilin (Cell Signaling, Danvers, MA, Cat.No. 9061S), the apoptosis marker PARP (Cell Signaling, Cat.No. 9542), pRb (directed against phosphorylated serine 807/8, Cell Signaling, diluted 1:1000 in 1% non-fat dry milk in Tris buffered Saline/0.1% Tween-20), and β-Actin (Sigma, Cat.No. A5441, clone AC-15) were detected using HRP-conjugated anti-mouse or anti-rabbit antibodies, respectively (Dako, Glostrup, Denmark). Visualization was performed using an enhanced chemoluminescence detection system (Super Signal West Pico, Thermo Scientific, Rockford, IL).

### WST-1 proliferation assay

To test whether low expression levels of spinophilin influences cellular proliferation of BC cells, we measured the cellular growth rate by applying the WST-1 proliferation assay. A number of 2×10^4^ MCF-7 or SUM159 cells per well were seeded in a 96-well culture plate. Cells were grown in regular growth medium for 24 h or 48 h. WST-1 proliferation reagent (Roche Applied Science, Vienna, Austria) was applied according to the manufacturer's recommendations. After four hours colorimetric changes were measured using a SpectraMax Plus (Molecular Devices, Sunnyvale, CA) at a wavelength of 450 nm with a reference wavelength at 620 nm. Three independent experiments with each six technical replicates per cell line and time point were performed.

### xCELLigence system (cell growth, cell migration and invasion)

To monitor cellular growth in real-time with a second independent method, the xCELLigence DP device (RTCA; Roche Diagnostics Mannheim, Germany) was used. 10,000 transfected SUM159 cells were seeded in electronic microtiter plates (E-Plate VIEW 16; Roche Diagnostics) and measured for 77 hours with the xCELLigence system according to the user manual. Cell density measurements were performed in triplicates and signal detection was done every 15 minutes. Cell migration and invasion of stably transfected SUM159 were also assayed using the xCELLigence Real-Time Cell Analyzer. For the invasion assay CIM-plate-16 wells (Roche) were pre-coated with 20 μl of matrigel diluted 1:40 in growth medium for 0.5 h at 37°C. Afterwards 20,000 cells for the migration assay and 40,000 cells for the invasion assay were plated in each well in serum-free medium. The lower medium chamber contained growth medium with 10% FCS. Cells were allowed to settle for 30 min at room temperature before being placed in the RTCA in a humidified incubator at 37°C with 5% CO2. Measurements were performed every 15 minutes for 24 hours. Data acquisition and analyses were performed using the RTCA software (version 1.2, Roche Diagnostics). The cell index (CI) is derived from electrical impedance changes as the cells interact with interdigitated microelectrodes on the bottom of the E-plate. Three replicates of each cell line were performed.

### Anchorage-independent growth assay

The efficiency of colony formation of stably transfected SUM159 and MCF-7 cells in soft agar was determined by plating 2,500 cells in 1 ml of complete growth medium containing 0.35% low gelling temperature agarose (Sigma, Seelze, Germany) over 1.5 ml of growth medium containing 0.5% agar (Sigma) in a 35mm dish. Cells were cultured at 37°C and 5% CO_2_ for up to 4 weeks. Colonies were stained with 0.005% crystal violet (Sigma) in 25% methanol and the number of colonies was counted using a dissecting microscope.

### Mammosphere formation assay

To assess the effect of low spinophilin expression on the self-renewal capacity (mammosphere formation), we performed a spheroid growth model as previously described [22] with slight modifications. In detail, the adherent growing BC cell lines were dissociated into single cells using trypsin/EDTA and 2,000 single cells per well seeded in ultra-low attachment 6-well plates (Corning, NY, USA) using serum-free MEBM (Lonza, Basel, Switzerland) medium (SFM). SFM was supplemented with 1xB27 supplement (Gibco), 20 ng/ml human epidermal growth factor EGF (Peprotech, Hamburg, Germany), 10 ng/ml human basic fibroblast growth factor FGF (Peprotech), 20 IU/ml Heparin (Baxter, Vienna, Austria) and 1% antibiotic/antimycotic solution (Sigma-Aldrich). Mammospheres were observed and counted under a microscope 10 days later. Three independent experiments per cell line with each three technical replicates were performed.

### Tumor xenograft model

For tumor xenograft experiments, female, five week-old NOD/SCID/IL-2rγnull (NSG−) mice were obtained from Charles River Breeding Laboratories (Sulzfeld, Germany). Stably transfected shRNA spinophilin-silenced or scrambled control SUM159 cells were re-suspended in phosphate-buffered saline (PBS) and subcutaneously injected at a density of 1×10^7^ cells into the flanks of mice (n = 5 per group). Animals were sacrificed 16 days after injection and lung tissue was excised for histological analyses. Tissues were fixed in 4% buffered formaldehyde for 24 hours, paraffin-embedded and stained by hematoxilin-eosin (HE). Slides were assessed by an experienced pathologist (A.A), who was blinded according to the cells of origin. Three animals per group were randomly selected for pathological analyses. All animal work was done in accordance with a protocol approved by the Institutional Animal Care and Use Committee at the Austrian Federal Ministry for Science and Research (BMWF) (vote 66.010/25-III/3b/2013).

### Microarray gene expression analysis

To detect the most differentially expressed genes in spinophilin-silenced basal-like BC cells, total RNA was isolated using the miRNeasy Mini Kit (Qiagen) according to the manual instructions. RNA was checked on Bioanalyzer BA2100 (Agilent; Foster City, CA). Sample with RIN (RNA integrity number) >8 were taken for whole transcriptome analysis on Affymetrix Human Gene 2.0 ST mRNA Arrays (Affymetrix; Santa Clara, CA). In detail 250 ng total RNA was amplified with NuGen Applause WT-Amp Plus ST System (NuGEN Technologies, Inc; San Carlos, CA). The great benefit of NuGEN is the SPIA amplification, which is a linear isothermal amplification process (SPIA: single primer isothermal amplification). cDNA was purified by MinElute Reaction Cleanup Kit (Qiagen), measured by NanoDrop and quality checked on the BioAnalyzer BA2100 (Agilent; Foster City, CA) using the RNA 6000 Nano LabChip (Agilent). An examination of ~250ng generated ssDNA showed a fragment size 2000nt which was satisfying for further processing. Following fragmentation and labeling of 5μg ssDNA was accomplished by NuGEN Encore Biotin Module (NuGEN Technologies, Inc; San Carlos, CA) according to the user's manual. The hybridization cocktail was adjusted to the final concentration suggested by the NuGEN Encore Biotin Module. Hybridization time was set to 17h at 45°C while rotating in a hybridization oven as recommended. Washing and staining (GeneChip® HT hybridization, Wash and Stain Kit; Affymetrix) was done with the Affymetrix Genechip® fluidics station 450 according to the manual (protocol on fluidics station: FS450_0007). Arrays were scanned with the Affymetrix GeneChip scanner GCS3000.

Evaluation of the hybridization controls and pre-analysis was done with Affymetrix Expression Console EC 1.3.1. Hybridizations were done at the Division Core Facility Molecular Biology at the Centre of Medical Research at the Medical University of Graz.

For normalization and data analysis Partek Genomic Suite v6.6 software (Partek Inc; St Louis, MO) was used. We used the RMA approach (robust multi-chip average normalization) including background correction, quantile normalization across all arrays, median polished summarization and log transformed of expression values.

For statistical analysis a two-way ANOVA was performed between spinophilin-silenced and control cells in biological triplicates. Genes with *p*<0.05 and fold change of at least 1.5 were considered to be significantly de-regulated.

All microarray gene expression data have been deposited in the National Center for Biotechnology Information (NCBI)'s Gene Expression Omnibus (GEO) and are accessible through GEO Series accession number GSE61889.

### Statistical analysis

All data represent mean values of at least three independent experiments ± SEM (standard error of mean). Student t-test or non-parametric tests were used where appropriate. For all calculations, *p*<0.05 was considered as significant.

## SUPPLEMENTARY MATERIAL, FIGURES, TABLES


