# Supplementary material for: Low spinophilin expression enhances aggressive biological behavior of breast cancer
Source: Oncotarget. 2015 Mar 14;6(13):11191–202. doi: 10.18632/oncotarget.3586 (PMC4484449; doi:10.18632/oncotarget.3586)
Supplement: Supplementary file 1 [file oncotarget-06-11191-s001.pdf]

Low spinophilin expression enhances aggressive biological behavior of breast cancer

Supplementary Material

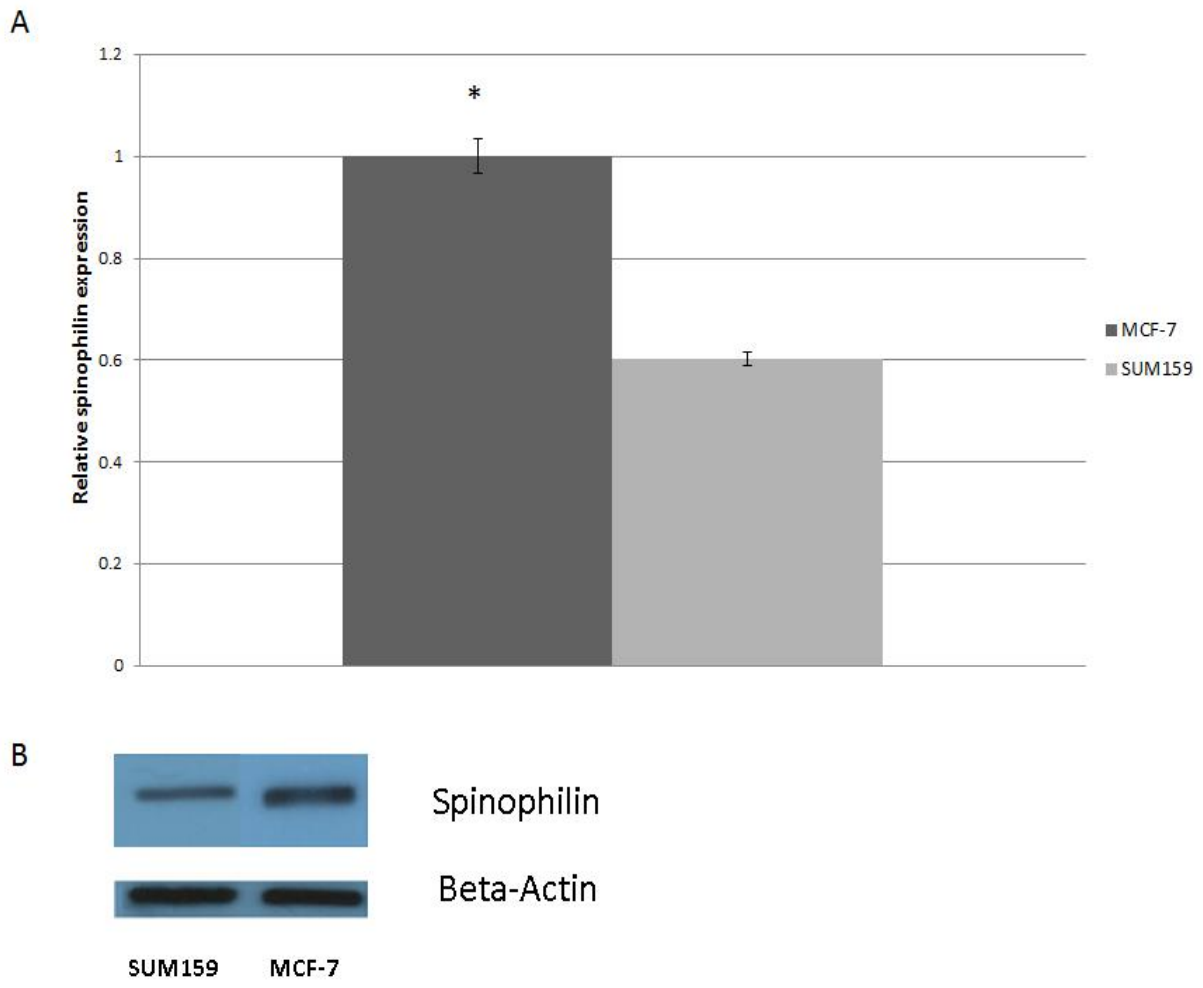

Supplementary Figure 1: Spinophilin expression in MCF-7 and SUM159 cells. The luminal A cell line MCF-7 shows higher spinophilin expression than the basal-like cell line SUM159 at mRNA **(A)** and protein **(B)** levels (1= SUM159, 2= MCF-7). Beta-actin was used as loading control.

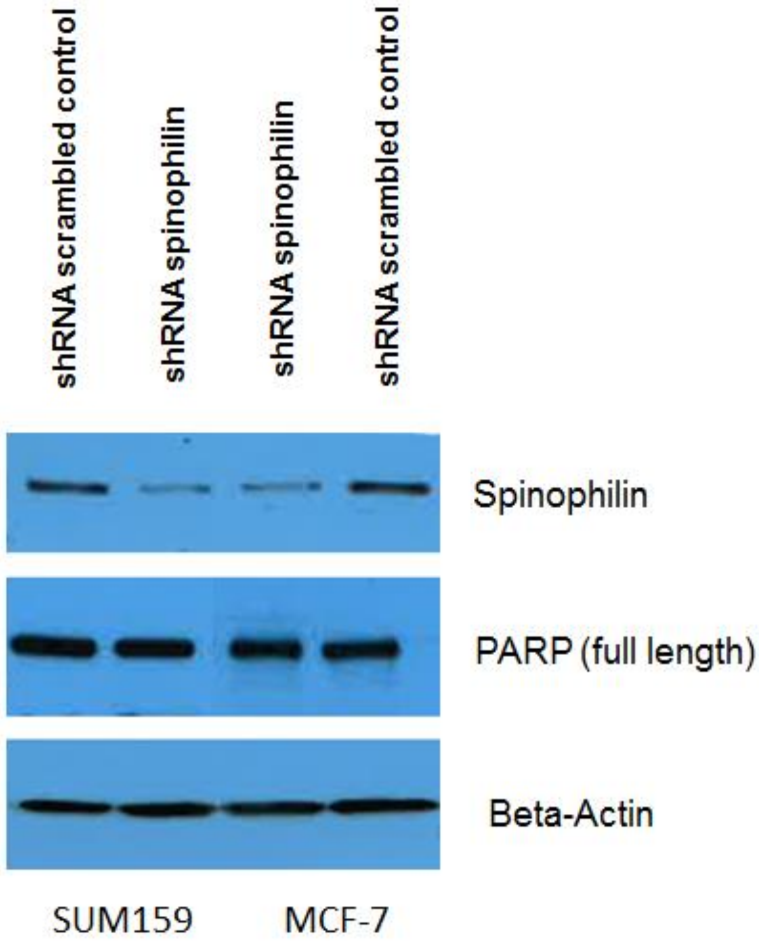

Supplementary Figure 2: Spinophilin expression and PARP detection in shRNA spinophilin-silenced breast cancer cell lines. Spinophilin protein levels are significantly reduced in both cell lines. For PARP staining, only the full length but not the cleaved PARP could be detected, indicating no differences in apoptosis rate. Beta-actin was used as loading control.

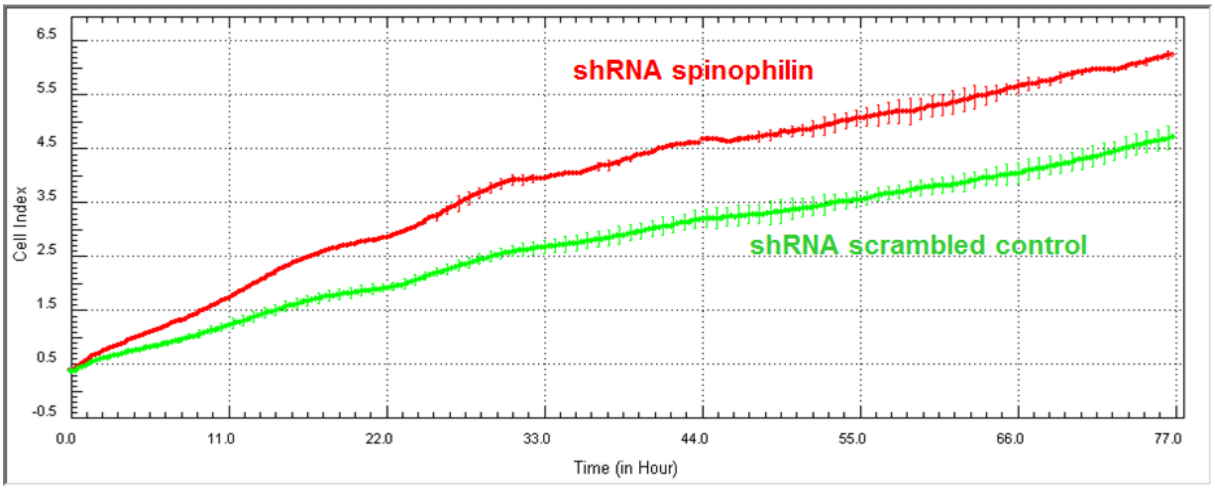

Supplementary Figure 3: Increased growth rate in spinophilin-silenced cells confirmed by an independent second method. Silencing of spinophilin increases cellular growth in SUM159 measured by xCELLigence. Real-time monitoring of the cell line SUM159 showed a significant higher cellular growth rates in spinophilin silenced cells (red) compared to control cells (green). The experiment was performed in triplicates and cell growth was monitored for 77 hours.

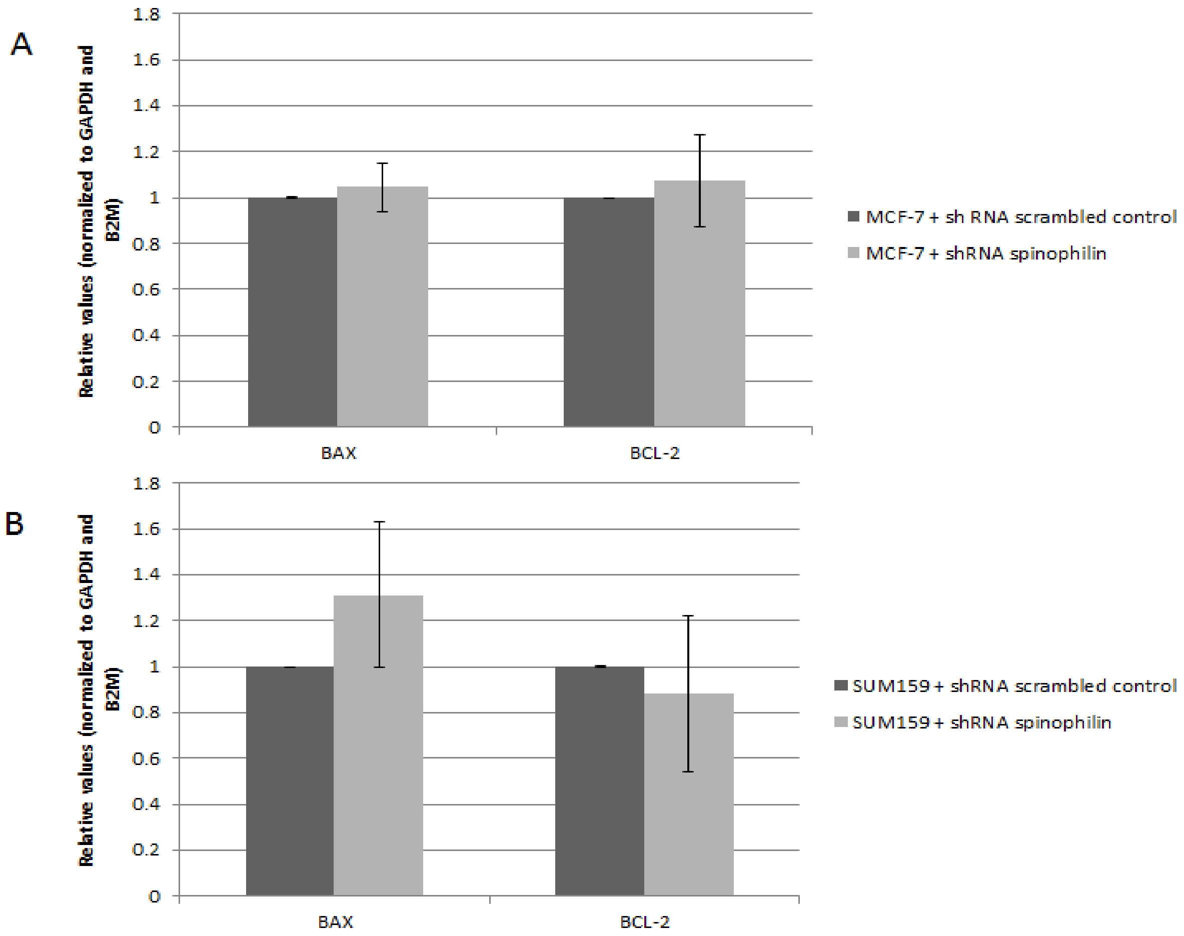

Supplementary Figure 4: Silencing of spinophilin does not affect apoptosis-related gene expression. Measurement of the pro-apoptotic Bax gene and the anti-apoptotic Bcl-2 gene by qRT-PCR showed no significant differences between spinophilin silenced cells and control cells. **A** shows the cell line MCF-7, **B** shows the cell line SUM159.

Supplementary Table 1: Gene specific primer sequences used for qRT-PCR (fw = forward primer, rv = reverse primer)

Gene/primer ID	sequence
Bcl-2_fw	GGAGGATTGTGGCCTTCTTTG
Bcl-2_rv	GCCGGTTCAGGTACTCAGTCAT
Bax_fw	CCTTTTCTACTTTGCCAGCAAAC
Bax_rv	GAGGCCGTCCCAACCAC
GAPDH_fw	AAGGTCGGAGTCAACGGATTT
GAPDH_rv	ACCAGAGTTAAAAGCAGCCCTG
B2M_fw	TGCTGTCTCCATGTTTGATGTATCT
B2M_rv	TCTCTGCTCCCCACCTCTAAGT
SSX1_fw	TGTCACCAACTGCTGCCAAC
SSX1_fv	CCAGGAGCAGTCTCACTTCAG
SSX2_fw	GGTGGAGCAGTCAGAACACA
SSX2_rv	CCCTTTTGGGTCCCTGTTGT
ITGBL1_fw	CCCAGGCTTCAGGAACTTCT
ITGBL1_rv	CCAGCTCCTCAGAGATGGC
RXFP2_fw	TCGCTCACAGTGCTGTTTCT
RXFP2_rv	GCTAGACAGATCCAGTTCTCCT
RYR2_fw	TGCATGAAAGCATCAAACGCA
RYR2_rv	TCCACCACACAGCCAATCTC
TSPAN7_fw	CATCGGAACTGGCACCACTA
TSPAN7_rv	TGGCATACAGTTTCAGCATCC
NEO1_fw	TCTTCCCACTGCCCATGTTC
NEO1_rv	TTCAGAGCTTGCTGGGACAG
CSTA_fw	ACATTAAGGTACGAGCAGGTG
CSTA_rv	ATGCTGCTAAAAGCCCGTCA
AMY1A_fw	CCTTCTGGGATGCTAGGCTG
AMY1A_rv	ATCTTGGCCAACGGTAGCTT
NAP1L3_fw	AGGCACACTGGGAACGAAAT
NAP1L3_rv	GAGATGTACCGCAGCCGAAT

Supplementary Table 2: The list of 30 top up- and down-regulated genes when comparing spinophilin-silenced SUM159 cells versus control cells.

Gene Symbol	RefSeq	Fold-Change (upregulated in SUM159 +spinophilin shRNA)
ITGBL1	NM_004791	6,40
SSX2	NM_003147	6,32
GAGE12C	NM_001098408	5,73
RXFP2	NM_130806	4,72
GAGE2A	NM_001127212	4,45
GAGE12H	NM_001098410	4,11
GAGE12G	NM_001098409	4,02
CXorf61	ENST00000371894	3,85
LOC400655	NR_034133	3,83
GAGE13	NM_001098412	3,62
PAGE1	NM_003785	3,53
GAGE12J	NM_001098406	3,34
NID1	NM_002508	3,24
SLC6A15	NM_182767	3,23
CXorf69	NM_001163438	3,16
LOC284801	NR_040095	3,06
C10orf136	NR_033923	3,00
MYBPC1	NM_001254718	2,96
RYR2	NM_001035	2,92
LOC100506823	XR_108334	2,90
SCN9A	NM_002977	2,88
SNORD93	NR_003075	2,85
SSX4	NM_005636	2,79
MST4	NM_016542	2,73
SEPP1	NM_001093726	2,68
LOC100287628	ENST00000562396	2,61
SSX4B	ENST00000376886	2,49
LOC100506835	NR_038268	2,48
FLJ37505	NR_033987	2,43
AMN1	NM_001113402	2,40

Gene Symbol	RefSeq	Fold-Change (downgulated in SUM159 +spinophilin shRNA)
LOC285505	AK129723	-2,69
TSPAN7	NM_004615	-2,59
LOC100505817	NR_038340	-2,53
NEO1	ENST00000339362	-2,40
CSTA	NM_005213	-2,37
AMY1A	NM_004038	-2,18
AMY2A	NM_000699	-2,18
ENKUR	NM_145010	-2,11
IL1A	NM_000575	-2,06
NAP1L3	NM_004538	-1,99
MUC15	ENST00000436318	-1,96
EPHA3	NM_005233	-1,91
XDH	NM_000379	-1,89
ELOVL4	NM_022726	-1,88
SH3BGRL2	NM_031469	-1,86
RN5S241	ENST00000391326	-1,86
PDE3B	NM_000922	-1,86
LOC643923	AK058066	-1,84
CYP2J2	ENST00000371204	-1,77
P2RY1	NM_002563	-1,75
VCAN	NM_004385	-1,75
SERPINB5	NM_002639	-1,74
NETO1	ENST00000327305	-1,73
SLCO5A1	NM_030958	-1,72
FAM59A	NM_001242409	-1,71
PPP4R4	NM_058237	-1,70
HEPH	ENST00000519389	-1,69
KRT75	ENST00000252245	-1,68
PTX3	NM_002852	-1,67
SNORA68	NR_000012	-1,66

Supplementary Table 3: Pathway analysis discovered several pathways changed.

		FC1.5 p0.005 up and down_233.txt 168 genes mapped (up and down)				FC1.5 p0.005 up_159.txt 110 genes mapped (up)				FC1.5 p0.005 down_74.txt 58 genes mapped (up)			
Pathways	no of genes hit against total (human)	genes within pathway	expected genes	expected direction	pvalue	genes within pathway	expected genes	expected direction	pvalue	genes within pathway	expected genes	expected direction	pvalue
Purine metabolism	7	2	0,05	+	1,40E-03	1	0,04	+	3,47E-02	1	0,02	+	1,85E-02
p53 pathway by glucose deprivation	21	2	0,16	+	1,17E-02	1	0,11	+	1,01E-01	1	0,06	+	5,44E-02
Sulfate assimilation	2	1	0,02	+	1,53E-02	1	0,01	+	1,00E-02	0	0,01	-	9,95E-01
Beta1 adrenergic receptor signaling pathway	30	2	0,23	+	2,28E-02	2	0,15	+	1,03E-02	0	0,08	-	9,23E-01
Beta2 adrenergic receptor signaling pathway	30	2	0,23	+	2,28E-02	2	0,15	+	1,03E-02	0	0,08	-	9,23E-01
Xanthine and guanine salvage pathway	3	1	0,02	+	2,29E-02	1	0,02	+	1,50E-02	0	0,01	-	9,92E-01
Oxytocin receptor mediated signaling pathway	43	2	0,33	+	4,40E-02	2	0,22	+	2,03E-02	0	0,11	-	8,92E-01
Thyrotropin-releasing hormone receptor signaling pathway	45	2	0,35	+	4,77E-02	2	0,23	+	2,20E-02	0	0,12	-	8,87E-01
Muscarinic acetylcholine receptor 1 and 3 signaling pathway	46	2	0,35	+	4,96E-02	2	0,23	+	2,30E-02	0	0,12	-	8,85E-01
5HT2 type receptor mediated signaling pathway	50	2	0,39	+	5,75E-02	2	0,25	+	2,68E-02	0	0,13	-	8,75E-01
Adenine and hypoxanthine salvage pathway	6	1	0,05	+	4,52E-02	0	0,03	-	9,70E-01	1	0,02	+	1,58E-02
Axon guidance mediated by semaphorins	19	1	0,15	+	1,36E-01	0	0,1	-	9,09E-01	1	0,05	+	4,93E-02
P53 pathway feedback loops 1	7	1	0,05	+	5,25E-02	0	0,04	-	9,65E-01	1	0,02	+	1,85E-02
p53 pathway	81	2	0,62	+	1,30E-01	0	0,41	-	6,64E-01	2	0,22	+	1,99E-02
